# Physician Wages in States with Expanded APRN Scope of Practice

**DOI:** 10.1155/2012/671974

**Published:** 2012-02-07

**Authors:** Patricia Pittman, Benjamin Williams

**Affiliations:** Department of Health Policy, School of Public Health and Health Services, The George Washington University, Washington, DC 20052, USA

## Abstract

In recent years, states have looked to reforms in advanced practice nursing scope of practice (SOP) barriers as a potential means to increase access to primary care while reducing costs. Currently, 16 states and the District of Columbia permit advanced practice registered nurses to practice independently of physicians, allowing them to perform functions such as diagnosing and prescribing under their own authority within the primary care setting. Given the resistance of many physician associations to these reforms, we asked whether the economic interests of primary care physicians might be affected by reforms. Using the Bureau of Labor Statistics data on earnings, we compared primary care physicians' earnings in states that have instituted SOP reforms to those that maintain these practice barriers. We also compared surgeons' earnings as a control group. Lastly, we compared the rate of growth in the earnings of primary care physicians and surgeons over the last ten years. This preliminary analysis revealed no evidence of differences in earnings across the two groups of states.

## 1. Background

In its 2010 report, “The future of nursing: leading change, advancing health,” the Institute of Medicine recommends the removal of scope of practice (SOP) barriers for advanced practice registered nurses (APRNs) such that they can “practice to the full extent of their education and training” (IOM, S-4). Currently, only 16 states and the District of Columbia (DC) allow APRNs to practice independently of physicians (For this study, we employ the standards of independent practice established by the Robert Wood Johnson Foundation Center to Champion Nursing in America [1], adapted from data collected for the annual Pearson Report [2]. By this standard, to be considered independent within a given state, APRNs must be permitted to diagnose and treat without physician supervision and must be permitted to prescribe medications either without physician supervision or with the signing of a one-time collaboration agreement with a physician or the state board of nursing (this may be for all prescribing or, in some states, only for prescribing of controlled substances, among which certain drugs may require case-specific consultations with a physician). In this way, our inclusion criteria for independent practice capture only states that grant APRNs complete or near-complete practice autonomy). In response to the IOM recommendation, the American Medical Association and the American Academy of Family Physicians, among others, have expressed their opposition, pointing out that physicians have more extensive education and arguing that nurses are not substitutable with primary care physicians [3, 4]. While the question of whether there are economic interests that might be negatively impacted by reforms is rarely discussed openly, the perceived impact of reform, particularly on primary care physicians, undoubtedly has and will continue to play a role in whether and to what extent SOP laws are reformed [5]. 

To the extent that there is a fear that physician income could decline as a result of expanding advanced practice nursing SOP, the assumption would be that there is a zero sum situation with regard to supply and demand in primary care and that, because APRNs are paid less for their services, payers will seek to substitute nurses in physician roles whenever possible, leading to reduced physician earnings and influence. Such assumptions do not account for unmet need, nor do they account for patient preferences, which may prioritize physician services when they are accessible, and sometimes even when care by a nurse is offered at a reduced cost and/or wait time [6].

There is, of course, a theoretical basis for believing that APRN services will increase competition and thus help contain costs. As an example of the effect, this idea has on policy, the Texas Legislative Budget Board has recently recommended the removal of the state's SOP barriers as a means of mitigating the state's substantial primary care shortage, as well as reducing costs to patients [7]. In their comments on two separate bills currently before the Texas State Senate designed to remove SOP barriers for APRNs, the U.S. Federal Trade Commission has expressed their support, emphasizing that the resulting increase in competition would serve the interests of health care consumers, and adding that their interpretation of the evidence suggests that that it would not adversely impact quality of care [8].

Containing costs through competition among providers is a complex proposition that we do not intend to address in this analysis. As a first attempt to explore whether earnings of employed primary care physicians in states with reforms could be negatively impacted, we compare their earnings to states that have not reformed their SOP laws. We also compare the wages of surgeons, a group unlikely to be impacted by expanded practices of APRNs, as a way to account for the myriad confounding factors that could affect wages across states. Finally, we compare the annual rate of change in wages over ten years (1999 to 2009) for all three practitioner groups to determine whether physician wages rise faster in states that retain SOP barriers.

## 2. Previous Research

Identifying empirical data to support or refute the presumed economic concerns of physicians is a complex task, both because the data is limited and because of the methodological challenge of attributing causality. In a literature review, only two studies focusing on the effect of SOP reforms on physician and nurse income were identified [9, 10]. Dueker et al. used information from the current population survey (CPS) from 1988 to 2002 for earnings data for APRNs, physician assistants (PAs), and physicians (MDs). The authors then constructed a binary “professional independence” variable based on annual rankings published in *Nurse Practitioner* and performed a regression analysis using this and the wage data. Perry employed a similar methodology, but using different data sources. For data on nurse practitioner (“NP,” a subset of APRNs) wages, he used the national sample survey of registered nurses (NSSRN), for Pas, he used the American Academy of Physician Assistants Annual Census (AAPAAC), and for MDs, he used the CPS. Perry used two binary variables to divide the regulatory landscape—prescriptive autonomy and reimbursement autonomy—and constructed two additional variables to measure the effect of time since reform on wages (on the assumption that changes in the labor market due to regulatory change build over time).

The two studies had varied results. Dueker et al. found that higher levels of professional autonomy were associated with a reduction in earnings of APRNs of 21% and an increase in PA earnings of 36%, with no statistically significant effect on the earnings of MDs. The authors propose three potential models of causation to explain this effect.

1. An increase in the supply of APRNs led to lower wages and greater practice autonomy through increased political influence of APRNs.
2. Regulatory changes attracted a greater number of APRNs to the state, causing wages to drop in response to increased supply.
3. There was a substitution effect wherein regulatory changes led to physicians hiring fewer APRNs and a greater number of PAs (who must work under physician supervision) for fear that their employing hospital or HMO would then shift some physician functions to APRNs.

While the available data did not allow the researchers to test these hypotheses directly, the lack of a significant correlation between changes in regulation and changes in wages leads the authors to suggest that the third explanation—that changes in physician hiring practices led to lower APRN and higher PA wages—is the most likely causal pathway.

In the second study, Perry found greater NP prescriptive autonomy to be associated with an increase in NP earnings by an average of 1.6% per year (for each year after authority was granted) and a decrease in PA and MD earnings of 1.4% and 7.6% per year, respectively. Greater NP payment authority was associated with a decrease in PA earnings of 0.3% per year, with no change in NP or MD earnings. Greater PA prescriptive authority was associated with a decrease in NP earnings of 0.8% per year, an increase in MD earnings of 8% per year, and no change in PA earnings. No significant relationship was observed between greater PA payment authority and earnings for any of the three groups. In the case of NP prescriptive autonomy, Perry suggests that the observed rise in NP wages and fall in MD and PA wages could be a result of the type of practitioner substitution described above, wherein an increase in the number of NPs providing primary care leads to lower MD market share, and thus lower wages. Interpreting the results on reimbursement autonomy, Perry suggests that, to the extent that NPs seek greater professional independence, demand for PAs rises to meet the demand for nonphysician caregivers within physician-led practices. This coupling between PA and MD earnings is also used to explain the observation that greater PA prescriptive autonomy is associated with greater MD earnings. Specifically, Perry suggests that the efficiency gained from allowing PAs greater autonomy benefit the practice in which PAs are employed, allowing those practices to employ fewer physicians, leading to lower operating costs. At the same time, reduced physician-led practice costs and increased PA practice autonomy lead to reduced demand for NPs, exerting downward pressure on NP earnings.

While only two studies looking at the direct impact of SOP laws on practitioner income were found, three additional studies were found that support the hypothesis that SOP reforms are associated with a greater supply of nurses in a state [11–13]. Sekscenski et al. looked at the supply of PAs, NPs, and certified nurse midwives (CNMs) and found a positive correlation between less restrictive nurse practice environments and nurse supply, in practitioners per capita. Two subsequent studies [12, 13] observed a similar correlation between supply of CNMs and less restrictive SOP laws.

A final study found in paper [14], an unpublished graduate thesis, looked at the growth in the concentration of NPs using county-level data across all states from 2001 to 2005 (without regard to local SOP laws). Using insurance payment data collected by the Agency for Healthcare Research and Quality, the author performed regression analyses to investigate the relationship between growth in NP concentration and the probability of being seen by a primary care MD (i.e., a family practitioner or pediatrician) when seeking primary care and the relationship between growth in NP concentration and earnings for both NPs and MDs. In counties undergoing high growth in NP concentration (defined as those within the top 25th percentile of NP growth over the study period), the author found that patients had 16.5% lower odds of seeing a family practitioner or pediatrician than in counties with lower NP growth. The author also found that high NP growth areas were associated with reimbursement rates 4.42% higher for NPs and 3.1% lower for primary care MDs. Both effects were statistically significant. The author concludes that higher NP concentrations do exert downward pressure on primary care MD wages via the substitution effect described above, but with the qualification that the relatively small number of NP observations available for analysis (some 1.59% of patient visits) and the relatively small magnitude of the impact on MD wages preclude this effect as being a driving force behind the recent shift away from primary and towards specialty care.

## 3. Methodology

In order to explore whether a simple association exists between primary care physician wages and expanded APRN SOP laws at the state level, we used 2009 annual wage data from the Bureau of Labor Statistics' (BLS) Occupational Employment Statistics Query System to compare three types of physician practitioners across states with and without expanded scope of practice laws [15].

It is important to note that BLS data includes only “employed” physicians, who under BLS definitions were approximately 70% of all practicing physicians in 2001 and 88% in 2008 [16, 17]. The percentage of self-employed physicians has been declining at a rate of approximately 2% per year for at least the past 25 years [17] (Different survey methodologies give different results with regard to physician employment. The CPS counts a physician as employed if he or she works any number of hours per week and thus includes a large number of physicians working in academic and administrative capacities. Surveys by other organizations (e.g., the Center for Studying Health System Change and the American Medical Association) exclude respondents working under a set number of hours or within particular specialties. Thus, the proportion of self-employed physicians given by the CPS is the lowest reported, as it includes the greatest number of employed physicians.)

In this analysis, we compare the earnings of primary care physicians (family and general practice physicians and general pediatricians) to the earnings of surgeons. We assume that because primary care physicians' practice overlaps with that of APRNs, in particular nurse practitioners, whereas surgeons' practices do not, any effect on earnings from increased nursing autonomy would appear among the former two groups without effecting surgeons' incomes. It is important to note that while a small number of APRNs may be certified as first assistants in surgery, this is not an area of independent APRN practice and, as such, would not be affected by variations in SOP laws.

There are indeed likely to be a variety of factors that differ between states that have implemented SOP reforms and those that have not. By using a comparison group within the profession and within the same state, however, we are able to account for a wide range of factors that might affect both practitioner groups, such as cost of living, insurance coverage rates in the state, physician density, and percent of population residing in rural areas.

For the comparison of states, we selected 14 of the 17 states (including DC) that instituted full SOP reform prior to the collection of the 2009 data and compared them to the remaining states. Of the 14 states considered, six had reforms prior to 1989, three in 1991, three in 1992, and two in 1993 (Table 1).

The average earnings of all three practitioner types in these states were compared to the remaining states that retain SOP barriers. We asked whether in states where nurses can practice independently of physicians, wages for family and general pediatrician physicians would be reduced relative to those of surgeons, as the latter category would not experience increased competition from nurses. Additionally, BLS data from 1999 (the earliest date for which wage data was available for these specific categories of practitioners) was used to calculate the annual rate of change in wages for all three practitioner groups.

## 4. Findings

**Figure 1:**
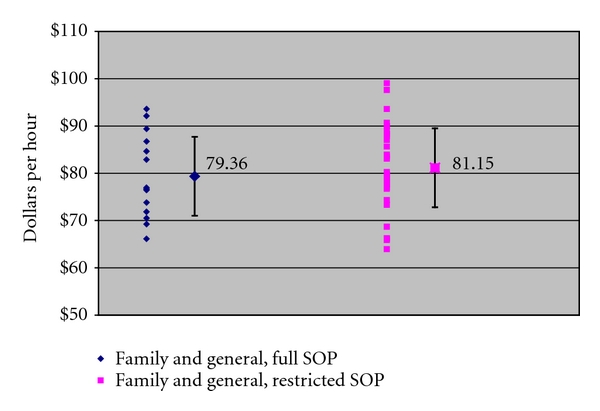
Family and general physician earnings, 2009 (with national average and standard deviation). Source: [16].

**Figure 2:**
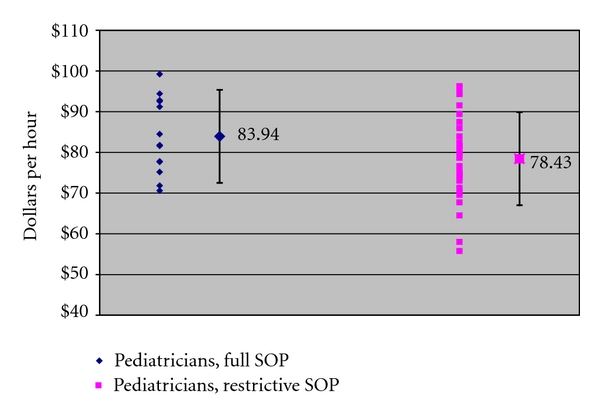
General pediatrician earnings in states with and without SOP barriers, 2009 (with national average and standard deviation). Source: [16].

**Figure 3:**
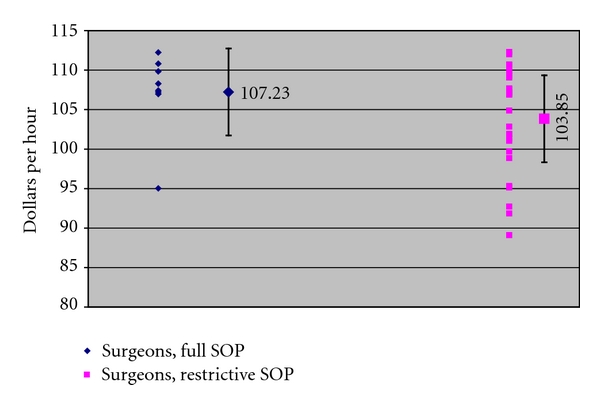
Surgeon earnings in states with and without SOP barriers, 2009 (with national average and standard deviation). Source: [16].

Figures 1, 2, and 3 present our principle findings, with average earnings and one standard deviation displayed by specialist type for each category of states in 2009. For family and general physicians (Figure 1), average earnings in states without SOP barriers (full SOP) were $79.36 per hour, and earnings in states with restrictive SOP laws were $81.15. For general pediatricians (Figure 2), earnings in full SOP states were $83.94 per hour, and earnings in restrictive SOP states were $78.43 per hour. For surgeons (Figure 3), earnings in full SOP states were $107.23 per hour, and earnings in restrictive SOP states were $103.85. In all three practitioner groups, average earnings in full and restrictive SOP states fell within one standard deviation of one another, confirming that, for each group, the variation in earnings between the two types of states is not statistically significant.

**Figure 4:**
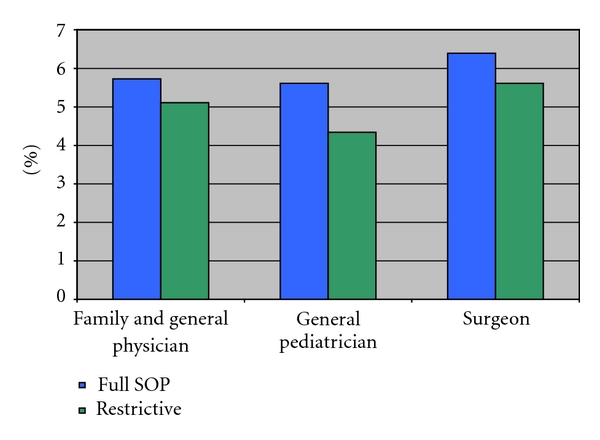
Average annual percent change in physician wages, 1999–2009. Source: [16].

The annual rate of change in physician earnings over ten years is presented in Figure 4. For family and general physicians, wages rose by 5.73% per year in full SOP states and 5.11% per year in restrictive SOP states. For general pediatricians, wages rose by 5.61% per year in full SOP states and 4.34% per year in restrictive SOP states. For surgeons, wages rose by 6.39% per year full SOP states and 5.61% per year in restrictive SOP states. These data again reveal no statistically significant differences between primary care physicians (family practice and physicians and general pediatricians), whose practice might be in competition with NPs in states with more liberal SOP laws, and that of surgeons, whose practice does not overlap with that of NPs.

## 5. Discussion

Our findings are descriptive and do not establish causality. However, they suggest that, at least among employed primary care physicians, expanded SOP laws do not impact their earnings. This differs from the findings of Dueker et al. and Perry, both of whom observed effects on both nurse and physician wages following the removal of SOP laws. These effects, however, could not be demonstrated to be a direct result of changes in SOP laws, and both the magnitude and direction of the effect were different between the two studies. In contrast, our analysis found no significant association between earnings in either direction for the physician groups compared. 

The rate of change calculations yielded did not reveal differences either. In fact, wages for all three practitioner groups rose at a slightly faster rate between 1999 and 2009 in states with more liberal SOP laws in states with restrictive laws. Where competition from NPs directly affecting the wages of MD via a substitution effect, one would expect to see the reverse—a slower rate of wage growth in states where nurses are allowed to compete for primary care dollars.

This preliminary analysis suggests that MD wages are not affected by changes in SOP barriers and/or that the removal of SOP barriers has a more nuanced effect on MD wages than simple economic substitution. For example, it is possible that, as APRNs assume a more independent role in primary care, other primary care providers begin to take on more complex and thus more costly cases. However, the fact that the same trends in wage growth were observed among surgeons as well as primary care physicians also suggests that they were due to factors unrelated to differences in SOP laws, as surgeon wages are unlikely to be affected by SOP laws.

This analysis relied on a comparison of wage data at just two points in time and did not control for other factors (e.g., NP supply changes, physician density, rates of insurance coverage, and local economic conditions). It is therefore possible that the true correlation between SOP laws and practitioner earnings was masked by confounders. In addition, it only provides data on employed physicians and excludes self-employed physicians. It also includes a number of employed physicians who work in research and academic settings, whose practice would not be “substitutable” with NPs.

To build upon this study, future work should identify and control for known confounders, employ additional years of data to further explore possible relationships between APRN SOP reform and practitioner wages, and employ more granular data sets to determine whether wages vary based on physician and practice characteristics.

## Figures and Tables

**Table 1:** Year of scope of practice reform.

Alaska	Prior to 1989
Arizona	Prior to 1989
Idaho	Prior to 1989
Oregon	Prior to 1989
Utah	Prior to 1989
Washington	Prior to 1989
DC	1991
Montana	1991
Rhode Island	1991
Iowa	1992
New Hampshire	1992
Wyoming	1992
Maine	1993
New Mexico	1993
Colorado*	2009
Hawaii*	2009
Maryland*	2010

*Not included in analysis.
